# On the Stress–Strength Reliability of Transmuted GEV Random Variables with Applications to Financial Assets Selection

**DOI:** 10.3390/e26060441

**Published:** 2024-05-23

**Authors:** Melquisadec Oliveira, Felipe S. Quintino, Dióscoros Aguiar, Pushpa N. Rathie, Helton Saulo, Tiago A. da Fonseca, Luan Carlos de Sena Monteiro Ozelim

**Affiliations:** 1 Department of Statistics, University of Brasília, Brasília 70910-900, DF, Brazil; melquisadec.oliveira@gmail.com (M.O.); felipes.quintino2@gmail.com (F.S.Q.); pushpanrathie@yahoo.com (P.N.R.); 2 Instituto de Ciências Exatas e Tecnológicas, Universidade Federal de Jataí, Jataí 75801-615, GO, Brazil; dioscoros.junior@ufj.edu.br; 3 Gama Engineering College, University of Brasília, Brasília 72444-240, DF, Brazil; fonsecafga@unb.br; 4 Department of Civil and Environmental Engineering, University of Brasília, Brasília 70910-900, DF, Brazil; luanoz@gmail.com

**Keywords:** stress–strength reliability, extreme-value ℍ-function, TGEV distribution, assets selection, 60E05, 62Exx, 62Fxx

## Abstract

In reliability contexts, probabilities of the type R=P(X<Y), where *X* and *Y* are random variables, have shown to be useful tools to compare the performance of these stochastic entities. By considering that both *X* and *Y* follow a transmuted generalized extreme-value (TGEV) distribution, new analytical relationships were derived for *R* in terms of special functions. The results hereby obtained are more flexible when compared to similar results found in the literature. To highlight the applicability and correctness of our results, we conducted a Monte-Carlo simulation study and investigated the use of the reliability measure P(X<Y) to select among financial assets whose returns were characterized by the random variables *X* and *Y*. Our results highlight that *R* is an interesting alternative to modern portfolio theory, which usually relies on the contrast of involved random variables by a simple comparison of their means and standard deviations.

## 1. Introduction

Consider a component with a strength *Y* and subjected to a stress *X*. The component fails if the stress *X* exceeds the component strength *Y*; otherwise, it works properly. For independent components, the stress–strength reliability (SSR) *R*, also referred to as stress–strength probability, is given by:

(1) $$\begin{array}{c} R=P\left(X<Y\right)=\int_{-\infty }^{+\infty }F_{X}\left(x\right)f_{Y}\left(x\right)dx, \end{array}$$

where $F_{X}$ and $f_{Y}$ denote, respectively, the cumulative distribution function (CDF) of *X* and the probability density function (PDF) of *Y*.

Although *R* was initially applied in the context of engineering, the interest in such metric spreads to several areas, such as household financial fragility [1], stock marketmodeling [2], asset selection [3], among others. We refer the reader to [4] for further details on stress–strength models.

The choice of an appropriate distribution to model both *X* and *Y* directly influences the calculation and estimation of R. In Finance, we have strong evidence that asset returns are better modeled by either α-stable processes (heavy-tailed alternative to Brownian motion [5]) or by heavy-tailed time series models [6,7]. The Extreme-Value Theory (EVT) made available a body of knowledge around heavy-tailed distributions, like the definition of the extreme-value distributions [8] (and its variations), which can be used as a proxy of various fat-tailed distributions. Several studies have successfully applied EVT to model financial data [3,9,10,11,12], where it has been shown that EVT-based models may provide adequate risk management strategies. Therefore, generalizations of extreme-value distributions may enhance the quality of the models, and this is the general contribution of the present work.

In this paper, we are interested in further exploring *R* calculations in the context of extreme-value distributions. The estimation of *R*, when *X* and *Y* are independent random variables following extreme-value distributions, has been extensively studied. For example, Ref. [13] derived the expression of *R* for the extreme distributions Gumbel, Fréchet, and Weibull, Ref. [14] considered a Bayesian analysis of the Fréchet stress–strength model, Ref. [15] discussed Bayesian estimation of P(Y<X) for the Weibull distribution with arbitrary parameters and [16] improved the estimation for *R* by not using transformations in the data and eliminating the constraints on the parameters in the case of the Weibull models. Closed-form expressions for *R* when *X* and *Y* follow generalized extreme-value (GEV) distributions were obtained in [3], who also proposed an estimation procedure for *R* by not using transformations in the data and with as few parameter restrictions as possible.

Several generalizations of the extreme-value distribution have been proposed, but in the present paper, the so-called transmuted generalized extreme-value (TGEV) distribution shall be considered. The TGEV distribution, initially proposed by [17], has since been extensively studied and applied in various modeling scenarios. Significant contributions to its application and understanding have been made by [18,19]. Essentially, the TGEV distribution is a modification of the generalized extreme-value distribution (GEV), whose CDF is given by:

(2) $$G\left(x\right)=\left\{\begin{array}{cc} \exp\left(-{\left(1+\gamma \frac{x-\mu }{\sigma }\right)}^{-1/\gamma }\right), & 1+\gamma \left(\frac{x-\mu }{\sigma }\right)>0\;\;\mathrm{and}\;\;\gamma \neq 0,\\ \exp\left(-\exp\left(-\frac{x-\mu }{\sigma }\right)\right), & x\in R\;\;\mathrm{and}\;\;\gamma =0, \end{array}\right.$$

where γ>0 is the shape parameter, μ∈R is the location parameter and σ>0 is the scale parameter. Then, the TGEV distribution is obtained as follows: given the GEV distribution G(x), the transmuted distribution function *F* is given by:

(3) $$\begin{array}{c} F\left(x\right)=\left(1+\lambda \right)G\left(x\right)-\lambda {\left[G\left(x\right)\right]}^{2},\;\left|\lambda \right|<1. \end{array}$$

Properties such as moments, quartiles, tail behavior, and order statistics, among others, were studied in [19]. They also showed its applicability in modeling log-returns of stock prices.

In [19], the TGEV parameters (μ,σ,γ,λ) were estimated by a maximum likelihood approach. In contrast, this work proposes a two-step estimation procedure. First, a GEV model is estimated to yield (μ,σ,γ) parameters. Then, a refinement step is taken by estimating the λ parameter in an attempt to improve the first step fit and to reduce the overall computational effort to estimate the TGEV parameters.

In this paper, we consider the problem of estimating the stress–strength parameter *R* when *X* and *Y* are independent TGEV random variables. In addition, our framework does not require transformations in the data and, to the best of our knowledge, allows for the fewest parameter restrictions.

Our main contributions are

- to derive *R* analytically in terms of special functions;
- to derive closed-form expressions for multicomponent system reliability;
- to propose an estimation procedure for *R* and validate such procedure via a simulation study and
- to apply the theoretical results in asset selection problems in finance.

The paper is organized as follows: in Section 2, we define the extreme-value H-function and the *H*-function, and we explicitly present the CDF and PDF of the TGEV distribution. Section 3 deals with the derivation of *R* when *X* and *Y* are independent TGEV random variables. The maximum likelihood estimation for *R* is presented in Section 4. In Section 5, we discuss a simulation study and a stock price modeling application for asset selection. The last section presents the conclusions.

## 2. Preliminaries

In this section, we give some definitions and results which will be used subsequently.

### 2.1. Special Functions

Recently, the extreme-value H-function was introduced in [20]. This function is defined as follows:

(4) $$H\left(a_{1},a_{2},a_{3},a_{4},a_{5},a_{6}\right):=\int_{0}^{\infty }y^{a_{6}}\exp\left(-a_{1}y-{\left(a_{2}y^{a_{3}}+a_{4}\right)}^{a_{5}}\right)dy,$$

where $ℜ\left(a_{1}\right),ℜ\left(a_{2}\right),ℜ\left(a_{4}\right)\in R_{+}$, and $a_{3},a_{5}\in C$. It is important to note that both $ℜ(a_{1})$ and $ℜ(a_{2})$ cannot be equal to zero simultaneously. Moreover, $ℜ(a_{6})>-1$ when $a_{1}\neq 0$, or when $a_{1}=0$ and $\mathrm{sign}\left(a_{3}\right)=\mathrm{sign}\left(a_{5}\right)$. Conversely, $ℜ(a_{6})<-1$ when $a_{1}=0$ and $\mathrm{sign}\left(a_{3}\right)\neq \mathrm{sign}\left(a_{5}\right)$. In this context, R, C, and *ℜ*, respectively, denote the set of real numbers, complex numbers, and the real part of a complex number.

Another important special function is the *H*-function, which can be defined by:

$$H_{p,q}^{m,n}\left[z|\begin{array}{c} \left(a_{1},A_{1}\right),\cdots ,\left(a_{p},A_{p}\right)\\ \left(b_{1},B_{1}\right),\cdots ,\left(b_{q},B_{q}\right) \end{array}\right]=\frac{1}{2\pi i}\int_{L}\frac{\prod_{k=1}^{m}\Gamma \left(b_{j}+B_{j}s\right)\prod_{j=1}^{n}\Gamma \left(1-a_{j}-A_{j}s\right)}{\prod_{k=m+1}^{q}\Gamma \left(1-b_{j}-B_{j}s\right)\prod_{j=n+1}^{p}\Gamma \left(a_{j}+A_{j}s\right)}z^{-s}ds,$$

where 0≤m≤q, 0≤n≤p (not both *m* and *n* zeros simultaneously), $A_{j}>0$ (j=1,⋯,p), $B_{k}>0$ (k=1,⋯,q), $a_{j}$ and $b_{k}$ are complex numbers such that no poles of $\Gamma (b_{k}+B_{k}s)$ (k=1,⋯,m) coincide with poles of $\Gamma (1-a_{j}-A_{j}s)$ (j=1,⋯,n). *L* is a suitable contour w−i∞ to w+i∞, w∈R, separating the poles of the two types mentioned above. For more details, see [21]. As special cases, we have:

(5) $$\int_{0}^{\infty }\exp\left\{-ay-by^{c}\right\}dy=\frac{1}{b^{1/c}c}H_{1,1}^{1,1}\left[\frac{a}{b^{1/c}}|\begin{array}{c} \left(\frac{c-1}{c},\frac{1}{c}\right)\\ \left(0,1\right) \end{array}\right]$$

for a>0, b>0 and c>0 and

(6) $$\int_{0}^{\infty }y^{d-1}\exp\left\{-ay-by^{c}\right\}dy=\frac{1}{a^{d}}H_{1,1}^{1,1}\left[ba^{-c}|\begin{array}{c} \left(1-d,c\right)\\ \left(0,1\right) \end{array}\right],$$

where a>0, b>0, c>0 and d>0.

### 2.2. Transmuted GEV Distribution

The CDF and the PDF of the TGEV distribution are given, respectively, by:

$$F\left(x;\mu ,\sigma ,\gamma ,\lambda \right)=\left\{\begin{array}{cc} \exp\left(-w^{-1/\gamma }\right)\left[\left(1+\lambda \right)-\lambda \exp\left(-w^{-1/\gamma }\right)\right], & \gamma \neq 0;\\ \exp\left(-\exp\left(-\left(\frac{w-1}{\gamma }\right)\right)\right)\left[\left(1+\lambda \right)-\lambda \exp\left(-\left(\frac{w-1}{\gamma }\right)\right)\right]\;, & \gamma =0, \end{array}\right.$$

and

(7) $$f\left(x;\mu ,\sigma ,\gamma ,\lambda \right)=\left\{\begin{array}{cc} \left[\frac{{\left(w\right)}^{-1-1/\gamma }\exp\left(-w^{-1/\gamma }\right)}{\sigma }\right]\left[\left(1+\lambda \right)-2\lambda \exp\left(-w^{-1/\gamma }\right)\right], & \gamma \neq 0;\\ \frac{\exp\left(-\left(\frac{w-1}{\gamma }\right)\right)\exp\left(-\exp(-\left(\frac{w-1}{\gamma }\right)\right)}{\sigma }\left[\left(1+\lambda \right)-2\lambda \exp\left(-\exp\left(-\left(\frac{w-1}{\gamma }\right)\right)\right)\right], & \gamma =0, \end{array}\right.$$

where w=1+(γ(x−μ)/σ) and supp(F)=supp(f)={x;w>0}.

Let $X∼TGEV(\mu_{x},\sigma_{x},\gamma_{x},\lambda_{x})$ and $Y∼TGEV(\mu_{y},\sigma_{y},\gamma_{y},\lambda_{y})$ be independent random variables. The stress–strength probability is given by:

$$\begin{array}{c} R=P\left(X<Y\right)=\int_{-\infty }^{+\infty }F_{X}\left(u;\mu_{x},\sigma_{x},\gamma_{x},\lambda_{x}\right)f_{Y}\left(u;\mu_{y},\sigma_{y},\gamma_{y},\lambda_{y}\right)du. \end{array}$$

**Figure 1:**
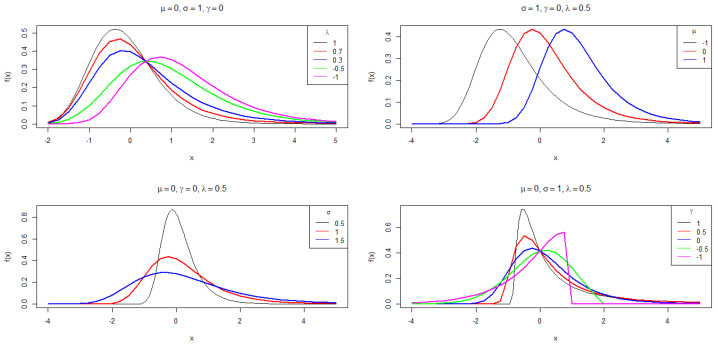
Plot for the TGEV PDF for some parameter choices.

In Section 5, we will apply the density *f* to modeling log-return stock prices. Furthermore, the support of *f* depends on the parameters. Thus, the maximum likelihood estimation is not as straightforward as in the usual cases. Figure 1 shows the behavior of the PDF of TGEV random variables for some choices of parameters. Proper parameter selection can be explored to represent the extremal models as particular cases of TGEV distribution, as shown in Table 1.

## 3. Main Results

In this section, the reliability of two independent TGEV random variables is derived in terms of H-functions. In addition, with suitable parameter restrictions, simpler expressions in terms of the H-function are also obtained. First, we consider the case of two independent TGEV with $\mathrm{sign}\left(\gamma_{x}\right)=\mathrm{sign}\left(\gamma_{y}\right)\neq 0$.

**Theorem** **1.** *Let X and Y be independent random variables, respectively, with distribution $TGEV(\mu_{x},\sigma_{x},\gamma_{x},\lambda_{x})$ and $TGEV(\mu_{y},\sigma_{y},\gamma_{y},\lambda_{y})$, $\mu_{j}\in R$, $\sigma_{j}\in R_{+}$, $\gamma_{j}\in R$$\left(\gamma_{j}\neq 0\right)$, $\lambda_{j}\in \left[-1,1\right]$, j∈{x,y}. Then*

- *When $\gamma_{j}>0$, j∈{x,y}:*

(8) $$\begin{array}{ccc} R=P(X<Y) & = & \left(1+\lambda_{x}\right)\left(1+\lambda_{y}\right)H\left(1,\frac{\gamma_{x}\sigma_{y}}{\gamma_{y}\sigma_{x}},-\gamma_{y},1+\frac{\gamma_{x}}{\sigma_{x}}\left(\mu_{y}-\mu_{x}-\frac{\sigma_{y}}{\gamma_{y}}\right),-\frac{1}{\gamma_{x}},0\right)\\ & & -2\lambda_{y}\left(1+\lambda_{x}\right)H\left(2,\frac{\gamma_{x}\sigma_{y}}{\gamma_{y}\sigma_{x}},-\gamma_{y},1+\frac{\gamma_{x}}{\sigma_{x}}\left(\mu_{y}-\mu_{x}-\frac{\sigma_{y}}{\gamma_{y}}\right),-\frac{1}{\gamma_{x}},0\right)\\ & & -\lambda_{x}\left(1+\lambda_{y}\right)H\left(1,2^{-\gamma_{x}}\frac{\gamma_{x}\sigma_{y}}{\gamma_{y}\sigma_{x}},-\gamma_{y},2^{-\gamma_{x}}\left[1+\frac{\gamma_{x}}{\sigma_{x}}\left(\mu_{y}-\mu_{x}-\frac{\sigma_{y}}{\gamma_{y}}\right)\right],-\frac{1}{\gamma_{x}},0\right)\\ & & +2\lambda_{x}\lambda_{y}H\left(2,2^{-\gamma_{x}}\frac{\gamma_{x}\sigma_{y}}{\gamma_{y}\sigma_{x}},-\gamma_{y},2^{-\gamma_{x}}\left[1+\frac{\gamma_{x}}{\sigma_{x}}\left(\mu_{y}-\mu_{x}-\frac{\sigma_{y}}{\gamma_{y}}\right)\right],-\frac{1}{\gamma_{x}},0\right), \end{array}$$

*provided that $\mu_{y}-\frac{\sigma_{y}}{\gamma_{y}}\geq \mu_{x}-\frac{\sigma_{x}}{\gamma_{x}}$. When $\mu_{y}-\frac{\sigma_{y}}{\gamma_{y}}<\mu_{x}-\frac{\sigma_{x}}{\gamma_{x}}$:*

(9) $$\begin{array}{ccc} R=P(X<Y) & = & 1-\left(1+\lambda_{y}\right)\left(1+\lambda_{x}\right)H\left(1,\frac{\gamma_{y}\sigma_{x}}{\gamma_{x}\sigma_{y}},-\gamma_{x},1+\frac{\gamma_{y}}{\sigma_{y}}\left(\mu_{x}-\mu_{y}-\frac{\sigma_{x}}{\gamma_{x}}\right),-\frac{1}{\gamma_{y}},0\right)\\ & & +2\lambda_{x}\left(1+\lambda_{y}\right)H\left(2,\frac{\gamma_{y}\sigma_{x}}{\gamma_{x}\sigma_{y}},-\gamma_{x},1+\frac{\gamma_{y}}{\sigma_{y}}\left(\mu_{x}-\mu_{y}-\frac{\sigma_{x}}{\gamma_{x}}\right),-\frac{1}{\gamma_{y}},0\right)\\ & & +\lambda_{y}\left(1+\lambda_{x}\right)H\left(1,2^{-\gamma_{y}}\frac{\gamma_{y}\sigma_{x}}{\gamma_{x}\sigma_{y}},-\gamma_{x},2^{-\gamma_{y}}\left[1+\frac{\gamma_{y}}{\sigma_{y}}\left(\mu_{x}-\mu_{y}-\frac{\sigma_{x}}{\gamma_{x}}\right)\right],-\frac{1}{\gamma_{y}},0\right)\\ & & -2\lambda_{y}\lambda_{x}H\left(2,2^{-\gamma_{y}}\frac{\gamma_{y}\sigma_{x}}{\gamma_{x}\sigma_{y}},-\gamma_{x},2^{-\gamma_{y}}\left[1+\frac{\gamma_{y}}{\sigma_{y}}\left(\mu_{x}-\mu_{y}-\frac{\sigma_{x}}{\gamma_{x}}\right)\right],-\frac{1}{\gamma_{y}},0\right). \end{array}$$

- *When $\gamma_{j}<0$, j∈{x,y}:*

(10) $$\begin{array}{ccc} R=P(X<Y) & = & \left(1+\lambda_{x}\right)\left(1+\lambda_{y}\right)H\left(1,\frac{\gamma_{x}\sigma_{y}}{\gamma_{y}\sigma_{x}},-\gamma_{y},1+\frac{\gamma_{x}}{\sigma_{x}}\left(\mu_{y}-\mu_{x}-\frac{\sigma_{y}}{\gamma_{y}}\right),-\frac{1}{\gamma_{x}},0\right)\\ & & -2\lambda_{y}\left(1+\lambda_{x}\right)H\left(2,\frac{\gamma_{x}\sigma_{y}}{\gamma_{y}\sigma_{x}},-\gamma_{y},1+\frac{\gamma_{x}}{\sigma_{x}}\left(\mu_{y}-\mu_{x}-\frac{\sigma_{y}}{\gamma_{y}}\right),-\frac{1}{\gamma_{x}},0\right)\\ & & -\lambda_{x}\left(1+\lambda_{y}\right)H\left(1,2^{-\gamma_{x}}\frac{\gamma_{x}\sigma_{y}}{\gamma_{y}\sigma_{x}},-\gamma_{y},2^{-\gamma_{x}}\left[1+\frac{\gamma_{x}}{\sigma_{x}}\left(\mu_{y}-\mu_{x}-\frac{\sigma_{y}}{\gamma_{y}}\right)\right],-\frac{1}{\gamma_{x}},0\right)\\ & & +2\lambda_{x}\lambda_{y}H\left(2,2^{-\gamma_{x}}\frac{\gamma_{x}\sigma_{y}}{\gamma_{y}\sigma_{x}},-\gamma_{y},2^{-\gamma_{x}}\left[1+\frac{\gamma_{x}}{\sigma_{x}}\left(\mu_{y}-\mu_{x}-\frac{\sigma_{y}}{\gamma_{y}}\right)\right],-\frac{1}{\gamma_{x}},0\right), \end{array}$$

*provided that $\mu_{y}-\frac{\sigma_{y}}{\gamma_{y}}<\mu_{x}-\frac{\sigma_{x}}{\gamma_{x}}$. When $\mu_{y}-\frac{\sigma_{y}}{\gamma_{y}}\geq \mu_{x}-\frac{\sigma_{x}}{\gamma_{x}}$:*

(11) $$\begin{array}{ccc} R=P(X<Y) & = & 1-\left(1+\lambda_{y}\right)\left(1+\lambda_{x}\right)H\left(1,\frac{\gamma_{y}\sigma_{x}}{\gamma_{x}\sigma_{y}},-\gamma_{x},1+\frac{\gamma_{y}}{\sigma_{y}}\left(\mu_{x}-\mu_{y}-\frac{\sigma_{x}}{\gamma_{x}}\right),-\frac{1}{\gamma_{y}},0\right)\\ & & +2\lambda_{x}\left(1+\lambda_{y}\right)H\left(2,\frac{\gamma_{y}\sigma_{x}}{\gamma_{x}\sigma_{y}},-\gamma_{x},1+\frac{\gamma_{y}}{\sigma_{y}}\left(\mu_{x}-\mu_{y}-\frac{\sigma_{x}}{\gamma_{x}}\right),-\frac{1}{\gamma_{y}},0\right)\\ & & +\lambda_{y}\left(1+\lambda_{x}\right)H\left(1,2^{-\gamma_{y}}\frac{\gamma_{y}\sigma_{x}}{\gamma_{x}\sigma_{y}},-\gamma_{x},2^{-\gamma_{y}}\left[1+\frac{\gamma_{y}}{\sigma_{y}}\left(\mu_{x}-\mu_{y}-\frac{\sigma_{x}}{\gamma_{x}}\right)\right],-\frac{1}{\gamma_{y}},0\right)\\ & & -2\lambda_{y}\lambda_{x}H\left(2,2^{-\gamma_{y}}\frac{\gamma_{y}\sigma_{x}}{\gamma_{x}\sigma_{y}},-\gamma_{x},2^{-\gamma_{y}}\left[1+\frac{\gamma_{y}}{\sigma_{y}}\left(\mu_{x}-\mu_{y}-\frac{\sigma_{x}}{\gamma_{x}}\right)\right],-\frac{1}{\gamma_{y}},0\right). \end{array}$$

**Proof.**  Set $S=\mathrm{supp}F_{X}\cap \mathrm{supp}f_{Y}$. Then

(12) $$S=\left\{\begin{array}{cc} (M,\infty ), & \mathrm{if}\mathrm{sign}\left(\gamma_{x}\right)=\mathrm{sign}\left(\gamma_{y}\right)=1,\\ (-\infty ,m), & \mathrm{if}\mathrm{sign}\left(\gamma_{x}\right)=\mathrm{sign}\left(\gamma_{y}\right)=-1,\\ R, & \mathrm{if}\mathrm{sign}\left(\gamma_{x}\right)=\mathrm{sign}\left(\gamma_{y}\right)=0, \end{array}\right.$$

where $M=\max\{\mu_{x}-\sigma_{x}/\gamma_{x},\mu_{y}-\sigma_{y}/\gamma_{y}\}$ and $m=\min\{\mu_{x}-\sigma_{x}/\gamma_{x},\mu_{y}-\sigma_{y}/\gamma_{y}\}$.

Note that

(13) $$\begin{array}{ccc} R=P(X<Y) & = & \int_{-\infty }^{\infty }F_{X}\left(u;\mu_{x},\sigma_{x},\gamma_{x},\lambda_{x}\right)f_{Y}\left(u;\mu_{y},\sigma_{y},\gamma_{y},\lambda_{y}\right)du\\ & = & \int_{S}\left(\exp\left(-w_{x}^{-1/\gamma_{x}}\right)\left[\left(1+\lambda_{x}\right)-\lambda_{x}\exp\left(-w_{x}^{-1/\gamma_{x}}\right)\right]\right.\\ & & \times \left.\left[\frac{{\left(w_{y}\right)}^{-1-1/\gamma_{y}}\exp\left(-w_{y}^{-1/\gamma_{y}}\right)}{\sigma_{y}}\right]\left[\left(1+\lambda_{y}\right)-2\lambda_{y}\exp\left(-w_{y}^{-1/\gamma_{y}}\right)\right]\right)du, \end{array}$$

where $w_{i}=1+\frac{\gamma_{i}}{\sigma_{i}}\left(u-\mu_{i}\right)$, i∈{x,y}. We have four cases to consider:

1. $\gamma_{x}>0$ and $\gamma_{y}>0$
  (a) $\mu_{y}-\sigma_{y}/\gamma_{y}\geq \mu_{x}-\sigma_{x}/\gamma_{x}$;
  (b) $\mu_{y}-\sigma_{y}/\gamma_{y}<\mu_{x}-\sigma_{x}/\gamma_{x}$;
2. $\gamma_{x}<0$ and $\gamma_{y}<0$
  (a) $\mu_{y}-\sigma_{y}/\gamma_{y}<\mu_{x}-\sigma_{x}/\gamma_{x}$;
  (b) $\mu_{y}-\sigma_{y}/\gamma_{y}\geq \mu_{x}-\sigma_{x}/\gamma_{x}$.

Let us consider case 1(a). Substituting $v=w_{y}^{-1/\gamma_{y}}$, it follows from (13) that

(14) $$\begin{array}{ccc} R & = & \left(1+\lambda_{x}\right)\left(1+\lambda_{y}\right)\int_{0}^{\infty }\exp\left(-v-{\left[\frac{\gamma_{x}\sigma_{y}}{\sigma_{x}\gamma_{y}}v^{-\gamma_{y}}+1+\frac{\gamma_{x}}{\sigma_{x}}\left(\mu_{y}-\mu_{x}-\frac{\sigma_{y}}{\gamma_{y}}\right)\right]}^{-1/\gamma_{x}}\right)dv\\ & & -2\left(1+\lambda_{x}\right)\lambda_{y}\int_{0}^{\infty }\exp\left(-2v-{\left[\frac{\gamma_{x}\sigma_{y}}{\sigma_{x}\gamma_{y}}v^{-\gamma_{y}}+1+\frac{\gamma_{x}}{\sigma_{x}}\left(\mu_{y}-\mu_{x}-\frac{\sigma_{y}}{\gamma_{y}}\right)\right]}^{-1/\gamma_{x}}\right)dv\\ & & -\lambda_{x}\left(1+\lambda_{y}\right)\int_{0}^{\infty }\exp\left(-v-2{\left[\frac{\gamma_{x}\sigma_{y}}{\sigma_{x}\gamma_{y}}v^{-\gamma_{y}}+1+\frac{\gamma_{x}}{\sigma_{x}}\left(\mu_{y}-\mu_{x}-\frac{\sigma_{y}}{\gamma_{y}}\right)\right]}^{-1/\gamma_{x}}\right)dv\\ & & +2\lambda_{x}\lambda_{y}\int_{0}^{\infty }\exp\left(-2v-2{\left[\frac{\gamma_{x}\sigma_{y}}{\sigma_{x}\gamma_{y}}v^{-\gamma_{y}}+1+\frac{\gamma_{x}}{\sigma_{x}}\left(\mu_{y}-\mu_{x}-\frac{\sigma_{y}}{\gamma_{y}}\right)\right]}^{-1/\gamma_{x}}\right)dv. \end{array}$$

Therefore, (8) follows from (4) and (14). For case 1(b), it suffices to notice that P(X<Y)=1−P(Y<X) and apply the result in (8) with interchanged sub-indices. For cases 2(a) and 2(b), the same rationale can be applied, just noticing that in such cases, *x* mostly takes negative values.    □

**Remark** **1.** *Note that if we take $\lambda_{x}=\lambda_{y}=0$, X and Y are random variables with GEV distributions, then our Theorem 1 generalizes the Theorem 3.1 in [3].*

**Remark** **2.** *In a practical scenario, the estimates $\left({\hat{\mu }}_{x},{\hat{\sigma }}_{x},{\hat{\gamma }}_{x},{\hat{\lambda }}_{x},{\hat{\mu }}_{y},{\hat{\sigma }}_{y},{\hat{\gamma }}_{y},{\hat{\lambda }}_{y}\right)$ should be obtained. Then, if $\mathrm{sign}\left({\hat{\gamma }}_{x}\right)=\mathrm{sign}\left({\hat{\gamma }}_{y}\right)\neq 0$, the conditions $\mu_{y}-\frac{\sigma_{y}}{\gamma_{y}}\geq \mu_{x}-\frac{\sigma_{x}}{\gamma_{x}}$ or $\mu_{y}-\frac{\sigma_{y}}{\gamma_{y}}<\mu_{x}-\frac{\sigma_{x}}{\gamma_{x}}$ must be verified and the corresponding R expression should be used.*

**Remark** **3.** *It follows from (5) that if $\mu_{x}-\sigma_{x}/\gamma_{x}=\mu_{y}-\sigma_{y}/\gamma_{y}$ and $\mathrm{sign}\left(\gamma_{x}\right)=\mathrm{sign}\left(\gamma_{y}\right)\neq 0$, then (8) can be written in terms of H-function as:*

(15) $$\begin{array}{ccc} R & = & \left(1+\lambda_{x}\right)\left(1+\lambda_{y}\right)\frac{\gamma_{x}}{\gamma_{y}}{\left(\frac{\sigma_{y}\gamma_{x}}{\sigma_{x}\gamma_{y}}\right)}^{1/\gamma_{y}}H_{1,1}^{1,1}\left[{\left(\frac{\gamma_{x}\sigma_{y}}{\gamma_{y}\sigma_{x}}\right)}^{1/\gamma_{y}}|\begin{array}{c} \left(1-\frac{\gamma_{x}}{\gamma_{y}},\frac{\gamma_{x}}{\gamma_{y}}\right)\\ \left(0,1\right) \end{array}\right]\\ & - & 2\lambda_{y}\left(1+\lambda_{x}\right)\frac{\gamma_{x}}{\gamma_{y}}{\left(\frac{\sigma_{y}\gamma_{x}}{\sigma_{x}\gamma_{y}}\right)}^{1/\gamma_{y}}H_{1,1}^{1,1}\left[2{\left(\frac{\gamma_{x}\sigma_{y}}{\gamma_{y}\sigma_{x}}\right)}^{1/\gamma_{y}}|\begin{array}{c} \left(1-\frac{\gamma_{x}}{\gamma_{y}},\frac{\gamma_{x}}{\gamma_{y}}\right)\\ \left(0,1\right) \end{array}\right]\\ & - & \lambda_{x}\left(1+\lambda_{y}\right)\frac{\gamma_{x}}{\gamma_{y}}2^{-\gamma_{x}/\gamma_{y}}{\left(\frac{\sigma_{y}\gamma_{x}}{\sigma_{x}\gamma_{y}}\right)}^{1/\gamma_{y}}H_{1,1}^{1,1}\left[2^{-\gamma_{x}/\gamma_{y}}{\left(\frac{\gamma_{x}\sigma_{y}}{\gamma_{y}\sigma_{x}}\right)}^{1/\gamma_{y}}|\begin{array}{c} \left(1-\frac{\gamma_{x}}{\gamma_{y}},\frac{\gamma_{x}}{\gamma_{y}}\right)\\ \left(0,1\right) \end{array}\right]\\ & + & \lambda_{x}\lambda_{y}\frac{\gamma_{x}}{\gamma_{y}}2^{1-\gamma_{x}/\gamma_{y}}{\left(\frac{\sigma_{y}\gamma_{x}}{\sigma_{x}\gamma_{y}}\right)}^{1/\gamma_{y}}H_{1,1}^{1,1}\left[2^{1-\gamma_{x}/\gamma_{y}}{\left(\frac{\gamma_{x}\sigma_{y}}{\gamma_{y}\sigma_{x}}\right)}^{1/\gamma_{y}}|\begin{array}{c} \left(1-\frac{\gamma_{x}}{\gamma_{y}},\frac{\gamma_{x}}{\gamma_{y}}\right)\\ \left(0,1\right) \end{array}\right]. \end{array}$$

*In particular, by using a special case of the H-function as seen in [21], if $\mu_{x}-\frac{\sigma_{x}}{\gamma_{x}}=\mu_{y}-\frac{\sigma_{y}}{\gamma_{y}}$, $\frac{\sigma_{y}}{\sigma_{x}}<2^{-\gamma_{x}}$ and $\gamma_{x}=\gamma_{y}\neq 0$, we have:*

(16) $$\begin{array}{ccc} R & = & \left(1+\lambda_{x}\right)\left(1+\lambda_{y}\right){\left[1+{\left(\frac{\sigma_{y}}{\sigma_{x}}\right)}^{-1/\gamma_{x}}\right]}^{-1}-2\left(1+\lambda_{x}\right)\lambda_{y}{\left[2+{\left(\frac{\sigma_{y}}{\sigma_{x}}\right)}^{-1/\gamma_{x}}\right]}^{-1}\\ & & -\lambda_{x}\left(1+\lambda_{y}\right){\left[1+2{\left(\frac{\sigma_{y}}{\sigma_{x}}\right)}^{-1/\gamma_{x}}\right]}^{-1}+\lambda_{x}\lambda_{y}{\left[1+{\left(\frac{\sigma_{y}}{\sigma_{x}}\right)}^{-1/\gamma_{x}}\right]}^{-1}. \end{array}$$

Lastly, we consider the cases of two independent TGEV distributions with $\gamma_{x}=\gamma_{y}=0$.

**Theorem** **2.** *Let $X∼TGEV(\mu_{x},\sigma_{x},0,\lambda_{x})$ and $Y∼TGEV(\mu_{y},\sigma_{y},0,\lambda_{y})$ be independent random variables with $\mu_{j}\in R$, $\sigma_{j}>0$, $\lambda_{j}\in \left[-1,1\right]$, j∈{x,y}. Then*

(17) $$\begin{array}{ccc} R & = & \left(1+\lambda_{x}\right)\left(1+\lambda_{y}\right)H\left(1,\exp\left(\frac{\mu_{x}-\mu_{y}}{\sigma_{x}}\right),\frac{\sigma_{y}}{\sigma_{x}},0,1,0\right)\\ & - & 2\left(1+\lambda_{x}\right)\lambda_{y}H\left(2,\exp\left(\frac{\mu_{x}-\mu_{y}}{\sigma_{x}}\right),\frac{\sigma_{y}}{\sigma_{x}},0,1,0\right)\\ & - & \lambda_{x}\left(1+\lambda_{y}\right)\exp\left(\frac{\mu_{x}-\mu_{y}}{\sigma_{x}}\right)H\left(1,\exp\left(\frac{\mu_{x}-\mu_{y}}{\sigma_{x}}\right),\frac{\sigma_{y}}{\sigma_{x}},0,1,\frac{\sigma_{y}}{\sigma_{x}}\right)\\ & + & 2\lambda_{x}\lambda_{y}\exp\left(\frac{\mu_{x}-\mu_{y}}{\sigma_{x}}\right)H\left(2,\exp\left(\frac{\mu_{x}-\mu_{y}}{\sigma_{x}}\right),\frac{\sigma_{y}}{\sigma_{x}},0,1,\frac{\sigma_{y}}{\sigma_{x}}\right). \end{array}$$

In particular, if we take $\sigma_{x}=\sigma_{y}$, we obtain the explicit form

$$R=\frac{\left(1+\lambda_{x}\right)\left(1+\lambda_{y}\right)}{1+\exp\left(\frac{\mu_{x}-\mu_{y}}{\sigma_{x}}\right)}-\frac{2\left(1+\lambda_{x}\right)\lambda_{y}}{2+\exp\left(\frac{\mu_{x}-\mu_{y}}{\sigma_{x}}\right)}-\frac{\lambda_{x}\left(1+\lambda_{y}\right)\exp\left(\frac{\mu_{x}-\mu_{y}}{\sigma_{x}}\right)}{{\left(1+\exp\left(\frac{\mu_{x}-\mu_{y}}{\sigma_{x}}\right)\right)}^{2}}+\frac{2\lambda_{x}\lambda_{y}\exp\left(\frac{\mu_{x}-\mu_{y}}{\sigma_{x}}\right)}{{\left(2+\exp\left(\frac{\mu_{x}-\mu_{y}}{\sigma_{x}}\right)\right)}^{2}}.$$

**Proof.**  Denote $F_{X}$ and $f_{Y}$, respectively, the CDF and PDF function of *X* and *Y*. Then

(18) $$\begin{array}{ccc} R & = & \int_{-\infty }^{+\infty }F_{X}\left(u;\mu_{x},\sigma_{x},\gamma_{x},\lambda_{x}\right)f_{Y}\left(u;\mu_{y},\sigma_{y},\gamma_{y},\lambda_{y}\right)du\\ & = & \int_{-\infty }^{+\infty }\exp\left(-\exp\left(-\frac{u-\mu_{x}}{\sigma_{x}}\right)\right)\left[\left(1+\lambda_{x}\right)-\lambda_{x}\exp\left(-\frac{u-\mu_{x}}{\sigma_{x}}\right)\right]\\ & \times & \frac{\exp\left(-\frac{u-\mu_{y}}{\sigma_{y}}\right)\exp\left(-\exp\left(-\frac{u-\mu_{y}}{\sigma_{y}}\right)\right)}{\sigma_{y}}\left[\left(1+\lambda_{y}\right)-2\lambda_{y}\exp\left(-\exp\left(-\frac{u-\mu_{y}}{\sigma_{y}}\right)\right)\right]du. \end{array}$$

Substituting $v=\exp\left(-\frac{u-\mu_{y}}{\sigma_{y}}\right)$, we can rewrite (18) as

(19) $$\begin{array}{ccc} R & = & \left(1+\lambda_{x}\right)\left(1+\lambda_{y}\right)\int_{0}^{\infty }\exp\left(-v-\exp\left(\frac{\mu_{x}-\mu_{y}}{\sigma_{x}}\right)v^{\sigma_{y}/\sigma_{x}}\right)dv\\ & - & 2\left(1+\lambda_{x}\right)\lambda_{y}\int_{0}^{\infty }\exp\left(-2v-\exp\left(\frac{\mu_{x}-\mu_{y}}{\sigma_{x}}\right)v^{\sigma_{y}/\sigma_{x}}\right)dv\\ & - & \lambda_{x}\left(1+\lambda_{y}\right)\exp\left(\frac{\mu_{x}-\mu_{y}}{\sigma_{x}}\right)\int_{0}^{\infty }v^{\sigma_{y}/\sigma_{x}}\exp\left(-v-\exp\left(\frac{\mu_{x}-\mu_{y}}{\sigma_{x}}\right)v^{\sigma_{y}/\sigma_{x}}\right)dy\\ & + & 2\lambda_{x}\lambda_{y}\exp\left(-\frac{\mu_{y}-\mu_{x}}{\sigma_{x}}\right)\int_{0}^{\infty }v^{\sigma_{y}/\sigma_{x}}\exp\left(-2v-\exp\left(\frac{\mu_{x}-\mu_{y}}{\sigma_{x}}\right)v^{\sigma_{y}/\sigma_{x}}\right)dv. \end{array}$$

Hence, (17) follows from (4) and (19).    □

**Remark** **4.** *It follows from (5) and (6) that (17) can be rewritten in terms of H-function as*

(20) $$\begin{array}{ccc} R & = & \left(1+\lambda_{x}\right)\left(1+\lambda_{y}\right)\frac{\sigma_{x}}{\sigma_{y}}\exp\left(\frac{\mu_{y}-\mu_{x}}{\sigma_{y}}\right)H_{1,1}^{1,1}\left[\exp\left(\frac{\mu_{y}-\mu_{x}}{\sigma_{y}}\right)|\begin{array}{c} \left(\frac{\sigma_{y}-\sigma_{x}}{\sigma_{y}},\frac{\sigma_{x}}{\sigma_{y}}\right)\\ \left(0,1\right) \end{array}\right]\\ & - & 2\left(1+\lambda_{x}\right)\lambda_{y}\frac{\sigma_{x}}{\sigma_{y}}\exp\left(\frac{\mu_{y}-\mu_{x}}{\sigma_{y}}\right)H_{1,1}^{1,1}\left[2\exp\left(\frac{\mu_{y}-\mu_{x}}{\sigma_{y}}\right)|\begin{array}{c} \left(\frac{\sigma_{y}-\sigma_{x}}{\sigma_{y}},\frac{\sigma_{x}}{\sigma_{y}}\right)\\ \left(0,1\right) \end{array}\right]\\ & - & \lambda_{x}\left(1+\lambda_{y}\right)\exp\left(\frac{\mu_{x}-\mu_{y}}{\sigma_{x}}\right)H_{1,1}^{1,1}\left[\exp\left(\frac{\mu_{x}-\mu_{y}}{\sigma_{x}}\right)|\begin{array}{c} \left(-\frac{\sigma_{y}}{\sigma_{x}},\frac{\sigma_{y}}{\sigma_{x}}\right)\\ \left(0,1\right) \end{array}\right]\\ & + & \lambda_{x}\lambda_{y}\exp\left(\frac{\mu_{x}-\mu_{y}}{\sigma_{x}}\right)2^{-\frac{\sigma_{y}}{\sigma_{x}}}H_{1,1}^{1,1}\left[2^{-\frac{\sigma_{y}}{\sigma_{x}}}\exp\left(\frac{\mu_{x}-\mu_{y}}{\sigma_{x}}\right)|\begin{array}{c} \left(-\frac{\sigma_{y}}{\sigma_{x}},\frac{\sigma_{y}}{\sigma_{x}}\right)\\ \left(0,1\right) \end{array}\right]. \end{array}$$

### Multicomponent System Reliability

Let $X_{1},\cdots ,X_{n}$ be independent and identically distributed random variables with distribution $TGEV(\mu_{x},\sigma_{x},\gamma_{x},\lambda_{x})$ and *Y* be an independent random variable with distribution $TGEV(\mu_{y},\sigma_{y},\gamma_{y},\lambda_{y})$. Set $M_{n}=\max\left\{X_{1},\cdots ,X_{n}\right\}$. Then,

$$P\left(M_{n}\leq u\right)=F^{n}\left(u;\mu_{x},\sigma_{x},\gamma_{x},\lambda_{x}\right)$$

and we have

(21) $$P\left(X_{1}<Y,\cdots ,X_{n}<Y\right)=P\left(M_{n}\leq Y\right)=\int_{-\infty }^{\infty }F^{n}\left(u;\mu_{x},\sigma_{x},\gamma_{x},\lambda_{x}\right)f\left(u;\mu_{y},\sigma_{y},\gamma_{y},\lambda_{y}\right)du=:I_{n}.$$

In a broader context, consider independent random variables $Y,X_{1},\cdots ,X_{k}$ with

$$Y∼TGEV\left(\mu_{y},\sigma_{y},\gamma_{y},\lambda_{y}\right)andX_{j}∼TGEV\left(\mu_{x},\sigma_{x},\gamma_{x},\lambda_{x}\right),j=1,\cdots ,k.$$

The reliability in a multicomponent stress–strength model is given by

$$\begin{array}{ccc} R_{s,k} & = & P(\mathrm{at}\;\mathrm{least}\;s\mathrm{of}\left(X_{1},\cdots ,X_{k}\right)\mathrm{exceed}Y)\\ & = & \sum_{j=s}^{k}\left(\begin{array}{c} k\\ j \end{array}\right)\int_{-\infty }^{\infty }{\left(1-F(u;\mu_{x},\sigma_{x},\gamma_{x},\lambda_{x})\right)}^{j}{\left(F(u;\mu_{x},\sigma_{x},\gamma_{x},\lambda_{x})\right)}^{k-j}f\left(u;\mu_{y},\sigma_{y},\gamma_{y},\lambda_{y}\right)du. \end{array}$$

Using a binomial expansion, we obtain

(22) $$R_{s,k}=\sum_{j=s}^{k}\sum_{r=0}^{j}\left(\begin{array}{c} k\\ j \end{array}\right)\left(\begin{array}{c} j\\ r \end{array}\right){\left(-1\right)}^{j-r}\int_{-\infty }^{\infty }{\left(F(u;\mu_{x},\sigma_{x},\gamma_{x},\lambda_{x})\right)}^{k-r}f\left(u;\mu_{y},\sigma_{y},\gamma_{y},\lambda_{y}\right)du.$$

Note that the integral terms in (22) is the same as (21) when n=k−r. Therefore,

$$R_{s,k}=\sum_{j=s}^{k}\sum_{r=0}^{j}\left(\begin{array}{c} k\\ j \end{array}\right)\left(\begin{array}{c} j\\ r \end{array}\right){\left(-1\right)}^{j-r}I_{k-r}.$$

Closed expressions for (21) are presented below.

**Theorem** **3.** *Let $X_{1},\cdots ,X_{n}$ be independent and identically distributed random variables with distribution $TGEV(\mu_{x},\sigma_{x},\gamma_{x},\lambda_{x})$ and Y be an independent random variable with distribution $TGEV(\mu_{y},\sigma_{y},\gamma_{y},\lambda_{y})$. Then*

- *When $\mathrm{sign}\left(\gamma_{x}\right)=\mathrm{sign}\left(\gamma_{y}\right)=1$:*

(23) $$I_{n}=\sum_{l=0}^{n}\left(\begin{array}{c} n\\ l \end{array}\right){\left(-1\right)}^{n-l}{\left(1+\lambda_{x}\right)}^{l}\lambda_{x}^{n-l}\left[\left(1+\lambda_{y}\right){\tilde{I}}_{n,l}-2\lambda_{y}{\hat{I}}_{n,l}\right],$$

*where*

$${\tilde{I}}_{n,l}=H\left(1,\frac{\gamma_{x}\sigma_{y}}{\gamma_{y}\sigma_{x}}{\left(2n-l\right)}^{-\gamma_{x}},-\gamma_{y},\left[1+\frac{\gamma_{x}}{\sigma_{x}}\left(\mu_{y}-\mu_{x}-\frac{\sigma_{y}}{\gamma_{y}}\right)\right]{\left(2n-l\right)}^{-\gamma_{x}},-\frac{1}{\gamma_{x}},0\right)$$

*and*

$${\hat{I}}_{n,l}=H\left(2,{\left(2n-l\right)}^{-\gamma_{x}}\frac{\gamma_{x}\sigma_{y}}{\gamma_{y}\sigma_{x}},-\gamma_{y},{\left(2n-l\right)}^{-\gamma_{x}}\left[1+\frac{\gamma_{x}}{\sigma_{x}}\left(\mu_{y}-\mu_{x}-\frac{\sigma_{y}}{\gamma_{x}}\right)\right],-\frac{1}{\gamma_{x}},0\right),$$

*provided that $\mu_{x}-\frac{\sigma_{x}}{\gamma_{x}}\leq \mu_{y}-\frac{\sigma_{y}}{\gamma_{y}}$.*

- *When $\mathrm{sign}\left(\gamma_{x}\right)=\mathrm{sign}\left(\gamma_{y}\right)=-1$*, (23) *holds provided that $\mu_{x}-\frac{\sigma_{x}}{\gamma_{x}}\geq \mu_{y}-\frac{\sigma_{y}}{\gamma_{y}}$.*
- *When $\gamma_{x}=\gamma_{y}=0$:*

(24) $$I_{n}=\sum_{l=0}^{n}\left(\begin{array}{c} n\\ l \end{array}\right){\left(-1\right)}^{n-l}{\left(1+\lambda_{x}\right)}^{l}\lambda_{x}^{n-l}\exp\left(\frac{\left(n-l\right)\left(\mu_{x}-\mu_{y}\right)}{\sigma_{x}}\right)\left[\left(1+\lambda_{y}\right){\tilde{J}}_{n,l}-2\lambda_{y}{\hat{J}}_{n,l}\right],$$

*where*

$${\tilde{J}}_{n,l}=H\left(1,n\exp\left(\frac{\mu_{x}-\mu_{y}}{\sigma_{x}}\right),\frac{\sigma_{y}}{\sigma_{x}},0,1,\frac{\left(n-l\right)\sigma_{y}}{\sigma_{x}}\right)$$

*and*

$${\hat{J}}_{n,l}=H\left(2,n\exp\left(\frac{\mu_{x}-\mu_{y}}{\sigma_{x}}\right),\frac{\sigma_{y}}{\sigma_{x}},0,1,\frac{\left(n-l\right)\sigma_{y}}{\sigma_{x}}\right).$$

**Proof.**  For simplicity of notations, denote

$$\begin{array}{cc} & F_{x}\left(u\right)=F\left(u;\mu_{x},\sigma_{x},\gamma_{x},\lambda_{x}\right),\\ & F_{y}\left(u\right)=F\left(u;\mu_{y},\sigma_{y},\gamma_{y},\lambda_{y}\right),\\ & G_{x}\left(u\right)=G\left(u;\mu_{x},\sigma_{x},\gamma_{x},\lambda_{x}\right), \end{array}$$

and

$$G_{y}\left(u\right)=G\left(u;\mu_{y},\sigma_{y},\gamma_{y},\lambda_{y}\right).$$

It follows from (3) and (21) that

$$\begin{array}{cc} I_{n} & =\int_{-\infty }^{+\infty }F_{x}^{n}\left(u\right)f_{y}\left(u\right)du\\ & =\int_{-\infty }^{+\infty }{\left[\left(1+\lambda_{x}\right)G_{x}\left(u\right)-\lambda_{x}G_{x}^{2}\left(u\right)\right]}^{n}f_{y}\left(u\right)du. \end{array}$$

By binomial expansion

(25) $$I_{n}=\sum_{l=0}^{n}\left(\begin{array}{c} n\\ l \end{array}\right){\left(-1\right)}^{n-l}{\left(1+\lambda_{x}\right)}^{l}\lambda_{x}^{n-l}\int_{-\infty }^{+\infty }G_{x}{\left(u\right)}^{2n-l}f_{y}\left(u\right)du.$$

Observe that $f_{y}\left(u\right)=F_{y}^{\prime }\left(u\right)$, which implies

(26) $$\begin{array}{ccc} \int_{-\infty }^{+\infty }G_{x}{\left(u\right)}^{2n-l}f_{y}\left(u\right)du & = & \int_{-\infty }^{+\infty }G_{x}{\left(u\right)}^{2n-l}\left[\left(1+\lambda_{y}\right)g_{y}\left(u\right)-2\lambda_{y}G_{y}\left(u\right)g_{y}\left(u\right)\right]du\\ & = & \left(1+\lambda_{y}\right)\int_{-\infty }^{+\infty }G_{x}{\left(u\right)}^{2n-l}g_{y}\left(u\right)du\\ & & -2\lambda_{y}\int_{-\infty }^{+\infty }G_{x}{\left(u\right)}^{2n-l}G_{y}\left(u\right)g_{y}\left(u\right)du. \end{array}$$

If $\mathrm{sign}\left(\gamma_{x}\right)=\mathrm{sign}\left(\gamma_{y}\right)=1$, it follows from (3.15) in [3] that

(27) $$\begin{array}{ccc} & & \int_{-\infty }^{+\infty }G_{x}{\left(u\right)}^{2n-l}g_{y}\left(u\right)du\\ & & =H\left(1,\frac{\gamma_{x}\sigma_{y}}{\gamma_{y}\sigma_{x}}{\left(2n-l\right)}^{-\gamma_{x}},-\gamma_{y},\left[1+\frac{\gamma_{x}}{\sigma_{x}}\left(\mu_{y}-\mu_{x}-\frac{\sigma_{y}}{\gamma_{y}}\right)\right]{\left(2n-l\right)}^{-\gamma_{x}},-\frac{1}{\gamma_{x}},0\right), \end{array}$$

provided that $\mu_{x}-\frac{\sigma_{x}}{\gamma_{x}}\leq \mu_{y}-\frac{\sigma_{y}}{\gamma_{y}}$. If $\mathrm{sign}\left(\gamma_{x}\right)=\mathrm{sign}\left(\gamma_{y}\right)=-1$, (3.17) in [3] implies (27) since $\mu_{x}-\frac{\sigma_{x}}{\gamma_{x}}\geq \mu_{y}-\frac{\sigma_{y}}{\gamma_{y}}$.

Observe that the integration range can be simplified using the results for the intersection of the supports of $G_{x}$ and $g_{y}$, such that:

$$S=\mathrm{supp}G_{x}\cap \mathrm{supp}g_{y}=\left\{\begin{array}{cc} \left(M,+\infty \right), & \gamma_{x}>0\;\mathrm{and}\;\gamma_{y}>0,\\ (-\infty ,m), & \gamma_{x}<0\;\mathrm{and}\;\gamma_{y}<0,\\ R, & \gamma_{x}=\gamma_{y}=0, \end{array}\right.$$

where $M=\max\{\mu_{x}-\frac{\sigma_{x}}{\gamma_{y}},\mu_{y}-\frac{\sigma_{y}}{\gamma_{y}}\}$ and $m=\min\{\mu_{x}-\frac{\sigma_{x}}{\gamma_{y}},\mu_{y}-\frac{\sigma_{y}}{\gamma_{y}}\}$. Then, if $\gamma_{x}>0$ and $\gamma_{y}>0$ (case $\gamma_{y}<0$ and $\gamma_{y}<0$ is analogous), we have that $\tilde{I}:=\int_{-\infty }^{+\infty }G_{x}{\left(u\right)}^{2n-l}G_{y}\left(u\right)g_{y}\left(u\right)du$ is given by

$$\begin{array}{ccc} \tilde{I} & = & \int_{M}^{+\infty }\exp\left\{-\left(2n-l\right){\left[1+\frac{\gamma_{x}}{\sigma_{x}}\left(u-\mu_{x}\right)\right]}^{-1/\gamma_{x}}-2{\left[1+\frac{\gamma_{y}}{\sigma_{y}}\left(u-\mu_{y}\right)\right]}^{-1/\gamma_{y}}\right\}\\ & & \times {\left[1+\frac{\gamma_{y}}{\sigma_{y}}\left(u-\mu_{y}\right)\right]}^{-1/\gamma_{y}-1}\frac{du}{\sigma_{y}}. \end{array}$$

Substituting $v={\left[1+\frac{\gamma_{y}}{\sigma_{y}}\left(u-\mu_{y}\right)\right]}^{-1/\gamma_{y}}$, we obtain

(28) $$\begin{array}{ccc} & & -2\lambda_{y}\int_{-\infty }^{+\infty }G_{x}{\left(u\right)}^{2n-l}G_{y}\left(u\right)g_{y}\left(u\right)du\\ & = & -2\lambda_{y}H\left(2,{\left(2n-l\right)}^{-\gamma_{x}}\frac{\gamma_{x}\sigma_{y}}{\gamma_{y}\sigma_{x}},-\gamma_{y},{\left(2n-l\right)}^{-\gamma_{x}}\left[1+\frac{\gamma_{x}}{\sigma_{x}}\left(\mu_{y}-\mu_{x}-\frac{\sigma_{y}}{\gamma_{x}}\right)\right],-\frac{1}{\gamma_{x}},0\right). \end{array}$$

Hence, (23) follows from (25), (26), (27) and (28). On the other hand, when $\gamma_{x}=\gamma_{y}=0$, the proof follows the same rationale as in the case of the proof of Theorem 2, just considering the binomial expansion in the process. This proof is omitted for simplicity.    □

## 4. Estimation

This section deals with parameter estimation for R=P(X<Y) given two independent TGEV random variables. The literature presents maximum likelihood estimators (MLEs) for *R* considering explicit forms of *R* obtained after severe parameter restrictions on extreme-value distributions (such as [14,15,22]). Those approaches require the estimation of the parameters to be done jointly in the two samples and require a series of transformations to be properly applied for TGEV components. For the TGEV distribution, we have two cases to consider: $\mathrm{sign}\left(\gamma_{x}\right)=\mathrm{sign}\left(\gamma_{y}\right)\neq 0$ and $\mathrm{sign}\left(\gamma_{x}\right)=\mathrm{sign}\left(\gamma_{y}\right)=0$. The first case requires $\mu_{y}-\sigma_{y}/\gamma_{y}\geq \mu_{x}-\sigma_{x}/\gamma_{x}$ or $\mu_{y}-\sigma_{y}/\gamma_{y}<\mu_{x}-\sigma_{x}/\gamma_{x}$ (Theorem 1). On the other hand, if $\gamma_{x}=\gamma_{y}=0$, we release any restrictions on the parameters for the expressions, as a single formula can be used to obtain *R* in terms of H functions (Theorem 2).

### 4.1. MLE for R

Let $X∼TGEV(\mu_{x},\sigma_{x},\gamma_{x},\lambda_{x})$ and $Y∼TGEV(\mu_{y},\sigma_{y},\gamma_{y},\lambda_{y})$ independent random variables with $\mathrm{sign}\left(\gamma_{x}\right)=\mathrm{sign}\left(\gamma_{y}\right)\neq 0$. Theorem 1 indicates that R=R(θ), where we denote $\theta =(\mu_{x},\sigma_{x},\gamma_{x},\lambda_{x},\mu_{y},\sigma_{y},\gamma_{y},\lambda_{y}).$ Thus, let $x=(X_{1},\cdots ,X_{n})$ be a random sample of $TGEV(\mu_{x},\sigma_{x},\gamma_{x},\lambda_{x})$ and consider an independent random sample $y=(Y_{1},\cdots ,Y_{m})$ of $TGEV(\mu_{y},\sigma_{y},\gamma_{y},\lambda_{y})$, with $\mathrm{sign}\left(\gamma_{x}\right)=\mathrm{sign}\left(\gamma_{y}\right)\neq 0$. Let $\hat{\theta }=\left({\hat{\mu }}_{x},{\hat{\sigma }}_{x},{\hat{\gamma }}_{x},{\hat{\lambda }}_{x},{\hat{\mu }}_{y},{\hat{\sigma }}_{y},{\hat{\gamma }}_{y},{\hat{\lambda }}_{y}\right)$ be the estimates of θ. Since Theorem 1 describes *R* in terms of integrals (hence continuous and measurable functions), we can estimate *R* simply as $\hat{R}=R\left(\hat{\theta }\right)$ due to the invariance property of MLE.

### 4.2. Parameters Estimation of TGEV Samples

Consider the PDF f(·;μ,σ,γ,λ) defined in (7). Take $x=(X_{1},\cdots ,X_{n})$ and $y=(Y_{1},\cdots ,Y_{m})$ independent random samples of sizes *n* and *m*, respectively. The likelihood function is given by:

(29) $$L\left(\theta ;x,y\right)=\prod_{j=1}^{n}f\left(X_{j};\mu_{x},\sigma_{x},\gamma_{x},\lambda_{x}\right)\prod_{i=j}^{m}f\left(Y_{j};\mu_{y},\sigma_{y},\gamma_{y},\lambda_{y}\right).$$

When $\gamma_{x}=\gamma_{y}=0$, the support of *f* does not depend on unknown parameters and the Theorem 2 does not require parameter restrictions. The log-likelihood function is given by

(30) $$\begin{array}{ccc} l(\theta ;x,y) & = & -n\sigma_{x}-m\sigma_{y}-\sum_{j=1}^{n}\frac{x_{j}-\mu_{x}}{\sigma_{x}}-\sum_{k=1}^{m}\frac{y_{k}-\mu_{y}}{\sigma_{y}}\\ & & -\sum_{j=1}^{n}\exp\left(-\frac{x_{j}-\mu_{x}}{\sigma_{x}}\right)+\sum_{j=1}^{n}\log\left[\left(1+\lambda_{x}\right)-2\lambda_{x}\exp\left(-\exp\left(-\frac{x_{j}-\mu_{x}}{\sigma_{x}}\right)\right)\right]\\ & & -\sum_{k=1}^{m}\exp\left(-\frac{y_{k}-\mu_{y}}{\sigma_{y}}\right)+\sum_{k=1}^{m}\log\left[\left(1+\lambda_{y}\right)-2\lambda_{y}\exp\left(-\exp\left(-\frac{y_{k}-\mu_{y}}{\sigma_{y}}\right)\right)\right]. \end{array}$$

Then, the MLE can be obtained by the log-likelihood function (30), equating its gradient to zero and finding its critical points.

When $\mathrm{sign}\left(\gamma_{x}\right)=\mathrm{sign}\left(\gamma_{y}\right)\neq 0$, the support of *f* depends on the unknown parameter (μ,σ,γ). Then, we are not able to obtain the MLE explicitly, so an additional numeric procedure is required to perform the likelihood maximization. This is similar to what happens with the GEV distribution (see [6] for a more detailed discussion). The likelihood function becomes:

(31) $$\begin{array}{ccc} L(\theta ;x,y) & = & \sigma_{x}^{-n}\sigma_{y}^{-m}\exp\left\{-\sum_{j=1}^{n}w_{x,j}^{-1/\gamma_{x}}\right\}\exp\left\{-\sum_{k=1}^{m}w_{y,k}^{-1/\gamma_{y}}\right\}\\ & \times & \prod_{j=1}^{n}\frac{\left[\left(1+\lambda_{x}\right)-2\lambda_{x}\exp\left(-w_{x,j}^{-1/\gamma_{x}}\right)\right]}{w_{x,j}^{1+1/\gamma_{x}}}1_{\left(0,\infty \right)}\left(w_{x,j}\right)\\ & \times & \prod_{k=1}^{m}\frac{\left[\left(1+\lambda_{y}\right)-2\lambda_{y}\exp\left(-w_{y,k}^{-1/\gamma_{y}}\right)\right]}{w_{y,k}^{1+1/\gamma_{y}}}1_{\left(0,\infty \right)}\left(w_{y,k}\right), \end{array}$$

where $w_{x,j}=1+\gamma_{x}\left(x_{j}-\mu_{x}\right)/\sigma_{x}$ and $w_{y,k}=1+\gamma_{y}\left(y_{k}-\mu_{y}\right)/\sigma_{y}$. Note that $\prod_{j=1}^{n}1_{\left(0,\infty \right)}\left(w_{x,j}\right)>0$ if and only if $w_{x,j}\in \left(0,\infty \right)$ for all j=1,⋯,n. A similar restriction should be observed for $w_{y,k}$. Numerical procedures must be applied to overcome the unavailability of an explicit MLE expression.

### 4.3. A Two-Step Estimation and Confidence Intervals

We introduce an alternative method for estimating θ through a two-step process outlined below:

**Example** **1.** *Given the samples x and y,*

***Step 1** We estimate $\left({\hat{\mu }}_{x},{\hat{\sigma }}_{x},{\hat{\gamma }}_{x}\right)$ and $\left({\hat{\mu }}_{y},{\hat{\sigma }}_{y},{\hat{\gamma }}_{y}\right)$ using MLE for the GEV (This estimation can be carried out utilizing the extRemes package within the R software version 4.3.3 [23].) distribution;*
***Step 2** The parameters $\left(\lambda_{x},\lambda_{y}\right)$ are estimated by determining*
$$\left({\hat{\lambda }}_{x},{\hat{\lambda }}_{y}\right)=\arg\max_{{\left[-1,1\right]}^{2}}L\left(\lambda_{x},\lambda_{y};x,y\right),$$
  *where $L(\lambda_{x},\lambda_{y};x,y)$ is derived from* (29) *using the estimated parameters from Step 1 as initial guesses.*

To choose between Theorems 1 and 2 to obtain $R(\hat{\theta })$, we need to verify if:

(a) ${\hat{\gamma }}_{x}\approx {\hat{\gamma }}_{y}\approx 0$;

or,

(b) ${\hat{\mu }}_{y}-{\hat{\sigma }}_{y}/{\hat{\gamma }}_{y}\geq {\hat{\mu }}_{x}-{\hat{\sigma }}_{x}/{\hat{\gamma }}_{x}$ or ${\hat{\mu }}_{y}-{\hat{\sigma }}_{y}/{\hat{\gamma }}_{y}<{\hat{\mu }}_{x}-{\hat{\sigma }}_{x}/{\hat{\gamma }}_{x}$.

Despite this additional verification, the computational time required for theExample 1 is expected to be less than that required for directly maximizing (29) and (30).

Example 2 describes the approach used in Section 5 to obtain confidence intervals (CIs) for the estimates of *R*.

**Example** **2.** *Let (x,y) be a sample of size n and M be a positive integer denoting the number of bootstrap repetitions.*

***Step 1** Generate bootstrap samples ${\left(x,y\right)}_{i}$.*
***Step 2** Compute the estimates $\hat{\theta_{i}}={\left({\hat{\mu }}_{x},{\hat{\sigma }}_{x},{\hat{\gamma }}_{x},{\hat{\lambda }}_{x},{\hat{\mu }}_{y},{\hat{\sigma }}_{y},{\hat{\gamma }}_{y},{\hat{\lambda }}_{y}\right)}_{i}$ based on ${\left(x,y\right)}_{i}$. In this case, the parameters of each bootstrap sample are individually estimated using Example 1.*
***Step 3** Obtain ${\hat{R}}_{i}=R\left({\hat{\theta }}_{i}\right)$ using Theorem 1 or 2.*
***Step 4** Repeat Steps 1 to 3 M times.*
***Step 5** The approximate 100(1−α)% confidence interval of $\hat{R}$ is given by $\left[{\hat{R}}_{M}\left(\alpha /2\right),{\hat{R}}_{M}\left(1-\alpha /2\right)\right]$, where ${\hat{R}}_{M}\left(\alpha \right)\approx {\hat{G}}^{-1}\left(\alpha \right)$ and $\hat{G}$ is the cumulative distribution function of $\hat{R}$.*

For the problem of asset selection using stress–strength reliability, only a single time series of observed returns is available for each asset. Then, the maximum likelihood estimation approach above is of utmost importance. To illustrate the suitability of the analytical closed-form expressions hereby derived, a simulation study is carried out in the next section. In such a case, several samples of size *n* can be drawn from each random variable, which is then used to estimate the value of *R* and can be repeated several times.

## 5. Applications

In this section, we provide a study involving Monte-Carlo simulations that analyze the performance of estimator $\hat{R}=R\left(\hat{\theta }\right)$. Additionally, we apply the stress–strength reliability model discussed in the preceding sections to actual real-world data.

### 5.1. Simulation Study

To evaluate the performance of the estimator $\hat{R}=R\left(\hat{\theta }\right)$, we fix several values of the parameters $\mu_{x}$,$\sigma_{x}$,$\gamma_{x}$, $\lambda_{x}$, $\mu_{y}$,$\sigma_{y}$,$\gamma_{y}$, $\lambda_{y}$, and then we generate N∈{100;1000;10,000} Monte-Carlo samples, each of which of size n=100, of the random variables $X∼TGEV(\mu_{x},\sigma_{x},\gamma_{x},\lambda_{x})$ and $Y∼TGEV(\mu_{y},\sigma_{y},\gamma_{y},\lambda_{y})$. We analyze the estimates $\hat{R}$, bias, and root mean squared error (RMSE).

As described by [19], random samples of TGEV distribution can be generated by the inversion method using the quantiles

$$F^{-1}\left(U\right)=\left\{\begin{array}{cc} \frac{\sigma }{\gamma }\left\{-1+{\left[-\log\left(\frac{1+\lambda -\sqrt{{\left(1+\lambda \right)}^{2}-4\lambda U}}{2\lambda }\right)\right]}^{-\gamma }\right\}, & \gamma \neq 0,\\ \mu +\sigma \left\{-\log\left[-\log\left(\frac{1+\lambda -\sqrt{{\left(1+\lambda \right)}^{2}-4\lambda U}}{2\lambda }\right)\right]\right\} & \gamma =0, \end{array}\right.$$

where *U* is a uniform random variable in [0,1].

For the simulation, for each line in the Table 2, Table 3, Table 4, Table 5 and Table 6 the following procedure was carried out:

(1) for each Monte-Carlo sample, the estimate $\hat{R}=R\left(\hat{\theta }\right)$ is computed;
(2) ${\hat{R}}_{MC}$ is evaluated by taking the sample mean of the Monte-Carlo samples $\hat{R}$;
(3) the bias is computed as the difference between the theoretical *R* value and ${\hat{R}}_{MC}$. The same applies to the root mean squared error, which also considers the true value as the analytically obtained one.

The TGEV distribution with negative-shape parameters is treated in Table 2, Table 4 and Table 6 (for N=100, 1000 and 10,000, respectively), while Table 3 and Table 5 deal with positive-shape parameters. In both cases, the estimator shows good behavior with minimal bias and low root mean squared error. Furthermore, it is clear that increasing the number of replications *N* leads to the same conclusions.

### 5.2. Real Data Set Application

Asset selection is addressed to evaluate the proposed framework. To guide the selection of financial assets when managing a portfolio, we adopt metrics of the type P(X<Y).

We start by modeling stock price log-returns as TGEV distributions, and afterward, we compare log-returns from tickers (companies) of different economic sectors and traded on BOVESPA (São Paulo Stock Exchange): BBAS3.SA (banking: Banco do Brasil S.A.), ITUB4.SA (banking: Itaú Unibanco Holding S.A.), VALE3.SA (mining: Vale S.A.) and VIIA3.SA (retail: Via Varejo S.A). From now on, we will omit the “.SA” suffix present on the tickers under analysis. The time series for each ticker represents the daily closing prices in Brazilian currency (R$, BRL) covering the period from 1 January 2022 to 30 April 2023. The analyzed data comprises a total of 331 daily prices.

**Figure 2:**
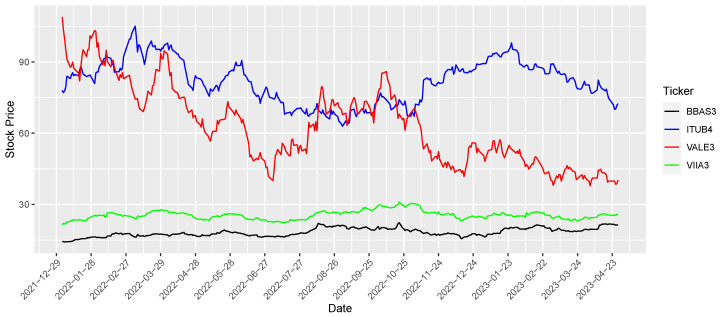
Daily closing values of stock prices for tickers BBAS3, ITUB4, VALE3, and VIIA3.

Figure 2 presents the stock prices for each ticker, highlighting their distinct value scales and volatility. Subsequently, we aim to compare the returns using the expression P(X<Y).

It is important to point out that these data sets were analyzed previously in the literature [3], and here we show that TGEV distribution fits the log-returns better than GEV, according to information criteria. The daily closing prices were imported directly through the software R by the command:

ticker = "BBAS3.SA"
quantmod::getSymbols(ticker, src = "yahoo", auto.assign = FALSE,
from = ’2022-01-01’, to = ’2023-04-30’, return.class = ’xts’).

**Figure 3:**
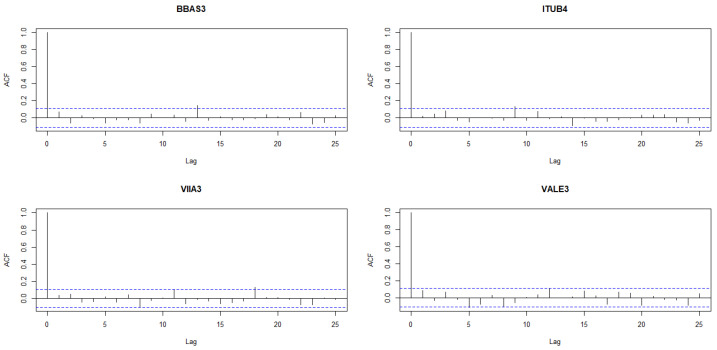
Log-returns autocorrelation function of log-returns (ACFs) for the assets BBAS3, ITUB4, VIIA3, and VALE3. The blue horizontal lines on the plots are the bounds ± 1.96sqrt(n).

We assume that the returns are independent. To meet independence requirements, we measure log-returns correlations using Pearson, Kendal, and Spearman methods comparing pairs of stocks that are not correlated. The pairs selected for comparison– VALE3-BBAS3, VALE3-ITUB4, and VALE3-VIIA3–all exhibited correlation measures equal to or below 0.25, as detailed in the Appendix of [3]. Figure 3 displays the autocorrelation function of log-returns, indicating an absence of discernible temporal correlations among the returns.

Descriptive statistics for the four data sets are presented in Table 7, showing certain symmetry of log-returns around zero and greater variability for VIIA3. The sample size was n=330 and each sample informs the daily closing stock price log-return. Across all data sets, the existence of extreme values is a consistent characteristic, which is in accordance with the nature of financial data.

Quintino et al. [3] showed that the GEV distribution adequately fits the data. Our interest lies in determining if the addition of the λ parameter provided by the TGEV distribution will improve the model fit. To accomplish this, we employed the two-step estimation method, described in theExample 1. Considering the different number of parameters of the GEV and TGEV models, to conduct a comparative analysis between these models, we utilized the information criteria Akaike Information Criterion (AIC), Bayesian Information Criterion (BIC), and Efficient Determination Criterion (EDC). Parameter estimates for the stock prices log-returns are presented in Table 8, while Table 9 shows that all criteria indicate that there was an improvement in the fit when using the TGEV distribution, compared to GEV.

**Figure 4:**
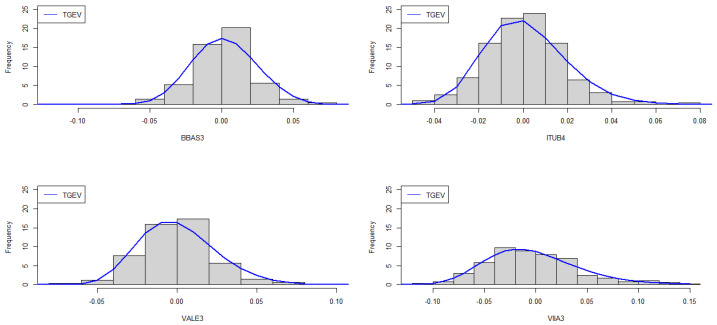
Histograms and fitted TGEV densities for the stock price log-returns.

**Figure 5:**
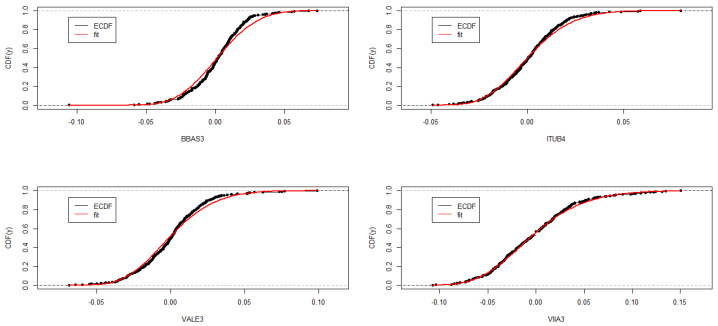
Fitted empirical CDF (ECDF) for TGEV models stock price log-returns.

**Figure 6:**
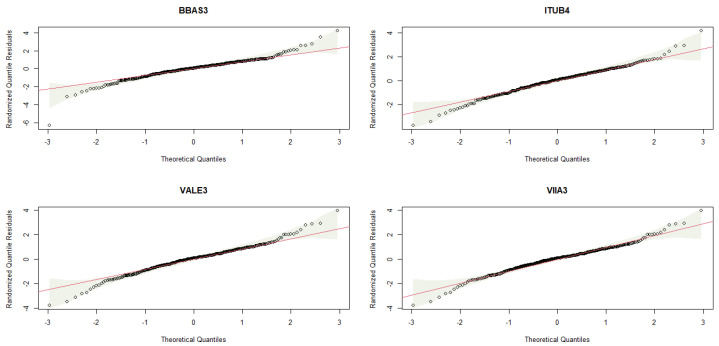
Normal Quantile-Quantile plot displaying residuals from fitted TGEV models.

The adequacy of the fitted TGEV distributions can be assessed through graphical evaluation methods. This includes plotting the theoretical PDF over the histogram (Figure 4), comparing the theoretical CDF against the empirical CDF (ECDF) (Figure 5), and examining the Normal Quantile-Quantile plots of the residuals (Figure 6). Although the Kolmogorov–Smirnov test rejects the TGEV adjustment for BBAS3 data, a visual examination of the histogram and ECDF might not discredit the suitability of the TGEV distribution. Furthermore, the Kolmogorov–Smirnov test tends to be overly sensitive, particularly for medium to large sample sizes, leading to its responsiveness even to minor deviations, which might account for this discrepancy.

Reliability measures, denoted as R=P(X<Y), play a pivotal role in an investor’s decision-making process. To simplify, when *X* and *Y* symbolize profit from log-returns and R<1/2, the investor tends to favor selecting the financial asset corresponding to *X*. Conversely, if R>1/2, the investor leans toward the opposite choice. However, when R=1/2, the decision becomes inconclusive. In this sense, Table 10 presents the estimates of P(X<Y) and the 95% Bootstrap confidence intervals, obtained by $\hat{R}$ and Example 2.

Utilizing the GEV distribution, reliability estimates ${\hat{R}}^{GEV}$ of 0.54, 0.54, and 0.43 for the VALE3-BBAS3, VALE3-ITUB4, and VALE3-VIIA3 pairs were obtained in [3], respectively. These values closely resembled those outlined in Table 10 for TGEV distribution. Regarding confidence intervals, there was a reduction in the interval size for the last pair, while the sizes remained consistent for the others.

Point estimates can also be compared with an empirical estimator that does not depend on the estimation of parameters or the choice of a probabilistic model. Let one consider the estimator:

$${\hat{R}}_{NP}=\frac{1}{n}\sum_{j=1}^{n}1_{\left\{x_{j}\leq y_{j}\right\}},$$

where $1_{A}$ denotes the indicator function on the set *A* and *n* is the sample size. The estimates obtained are, respectively, 0.55, 0.55, and 0.43 for the pairs presented in Table 10, showing the proximity of parametric and nonparametric estimates.

In Table 10, all the confidence intervals crossed the 0.5 edge; however, reliability measurement for the pair VALE3xVIIA3 brings some evidence that VALE3 should be the asset to be selected.

## 6. Conclusions

In this paper, we studied the stress–strength reliability R=P(X<Y) when both *X* and *Y* follow independent TGEV distributions. Thus, exact expressions for *R* have been obtained in terms of the extreme-value H-function with minimal parameter restrictions. With additional restrictions, it was shown that *R* can be calculated in terms of *H*-functions.

The present work evaluated the advantages yielded by adding a λ parameter to the GEV distribution and modelling data sets with the TGEV distribution. The added parameter brought a more complex analytical derivation of R=P(X<Y) and an expected increase in the computational effort to estimate it. To avoid the computational burden of an added parameter, we proposed a two-step estimation where we first fit a GEV model and then estimate the TGEV parameter λ. Notwithstanding the complexities of an added parameter, information criteria demonstrated the superiority of TGEV models when compared to GEV ones. This advantage is also perceived when estimating probabilities R=P(X<Y) by obtaining better estimates.

Monte-Carlo simulations attested to the performance of the analytical closed-form expressions hereby derived. By applying our methodology to real-world financial data, we could orient a stock selection procedure by calculating P(X<Y) when both *X* and *Y* represent stock returns. In summary, when *X* and *Y* represent the return of the stock prices and R<1/2, the investor should choose the variable *X*. If R>1/2, the opposite occurs. The case R=1/2 is inconclusive.

The framework we explored in this work can be a starting point to study probabilities R=P(X<Y) for recently proposed extreme-value distributions like bimodal Gumbell, bimodal Weibull, bimodal GEV, and extreme-value bivariate models. 

## Figures and Tables

**Table 1:** Particular cases of TGEV model.

Distribution	CDF
GEV	F(x;0,1,γ,0)
Fréchet	F((x−1)/γ;0,1,γ,0), γ>0
Reversed Weibull	F(−(1+x)/γ;0,1,γ,0), γ<0
Gumbel	F(x;0,1,0,0)

**Table 2:** Negative-shape mean. bias and root mean squared error (RMSE) of ${\hat{R}}_{MC}$(N=100andn=100).

$\mu_{x}$	$\sigma_{x}$	$\gamma_{x}$	$\lambda_{x}$	$\mu_{y}$	$\sigma_{y}$	$\gamma_{y}$	$\lambda_{y}$	*R*	${\hat{R}}_{\mathrm{MC}}$	Bias	RMSE
0	0.7	−0.1	0.3	0	0.5	−0.2	0.1	0.50945	0.50151	−0.00793	0.00030
0	0.7	−0.1	0.3	0.5	0.5	−0.2	0.1	0.71367	0.70374	−0.00993	0.00034
0	0.7	−0.1	0.3	0	0.7	−0.2	0.1	0.52511	0.51976	−0.00534	0.00031
0	0.7	−0.1	0.3	0.5	0.7	−0.2	0.1	0.70569	0.70066	−0.00503	0.00026
0	0.7	−0.1	0.3	0	0.5	−0.4	0.1	0.49137	0.48478	−0.00659	0.00029
0	0.7	−0.1	0.3	0.5	0.5	−0.4	0.1	0.69977	0.69183	−0.00794	0.00030
0	0.7	−0.1	0.3	0	0.7	−0.4	0.1	0.50809	0.50191	−0.00618	0.00030
0	0.7	−0.1	0.3	0.5	0.7	−0.4	0.1	0.69163	0.68617	−0.00546	0.00033
0	0.7	−0.1	0.3	0	0.5	−0.2	0.2	0.49602	0.48709	−0.00893	0.00036
0	0.7	−0.1	0.3	0.5	0.5	−0.2	0.2	0.70372	0.69562	−0.00810	0.00034
0	0.7	−0.1	0.3	0	0.7	−0.2	0.2	0.50870	0.50454	−0.00416	0.00029
0	0.7	−0.1	0.3	0.5	0.7	−0.2	0.2	0.69299	0.68870	−0.00429	0.00031
0	0.7	−0.1	0.3	0	0.5	−0.4	0.2	0.47863	0.47081	−0.00782	0.00030
0	0.7	−0.1	0.3	0.5	0.5	−0.4	0.2	0.68979	0.68537	−0.00442	0.00027
0	0.7	−0.1	0.3	0	0.7	−0.4	0.2	0.49230	0.49000	−0.00230	0.00033
0	0.7	−0.1	0.3	0.5	0.7	−0.4	0.2	0.67876	0.67348	−0.00529	0.00030

**Table 3:** Positive-shape mean. bias and root mean squared error (RMSE) of ${\hat{R}}_{MC}$(N=100andn=100).

$\mu_{x}$	$\sigma_{x}$	$\gamma_{x}$	$\lambda_{x}$	$\mu_{y}$	$\sigma_{y}$	$\gamma_{y}$	$\lambda_{y}$	*R*	${\hat{R}}_{\mathrm{MC}}$	Bias	RMSE
0	0.7	0.1	0.3	0	0.5	0.2	0.1	0.53183	0.52584	−0.00600	0.00030
0	0.7	0.1	0.3	0.5	0.5	0.2	0.1	0.71971	0.71210	−0.00761	0.00029
0	0.7	0.1	0.3	0	0.7	0.2	0.1	0.54076	0.53666	−0.00410	0.00030
0	0.7	0.1	0.3	0.5	0.7	0.2	0.1	0.71272	0.70832	−0.00440	0.00030
0	0.7	0.1	0.3	0	0.5	0.4	0.1	0.54736	0.54049	−0.00686	0.00029
0	0.7	0.1	0.3	0.5	0.5	0.4	0.1	0.73060	0.72135	−0.00926	0.00031
0	0.7	0.1	0.3	0	0.7	0.4	0.1	0.55478	0.55053	−0.00425	0.00023
0	0.7	0.1	0.3	0.5	0.7	0.4	0.1	0.72470	0.71852	−0.00618	0.00027
0	0.7	0.1	0.3	0	0.5	0.2	0.2	0.51767	0.51685	−0.00082	0.00028
0	0.7	0.1	0.3	0.5	0.5	0.2	0.2	0.71043	0.70462	−0.00581	0.00030
0	0.7	0.1	0.3	0	0.7	0.2	0.2	0.52393	0.52110	−0.00284	0.00028
0	0.7	0.1	0.3	0.5	0.7	0.2	0.2	0.70105	0.69495	−0.00610	0.00029
0	0.7	0.1	0.3	0	0.5	0.4	0.2	0.53280	0.52937	−0.00343	0.00031
0	0.7	0.1	0.3	0.5	0.5	0.4	0.2	0.72130	0.71108	−0.01021	0.00031
0	0.7	0.1	0.3	0	0.7	0.4	0.2	0.53775	0.53576	−0.00199	0.00027
0	0.7	0.1	0.3	0.5	0.7	0.4	0.2	0.71325	0.71132	−0.00193	0.00024

**Table 4:** Negative-shape mean, bias, and root mean squared error (RMSE) of ${\hat{R}}_{MC}$(N=1000andn=100).

$\mu_{x}$	$\sigma_{x}$	$\gamma_{x}$	$\lambda_{x}$	$\mu_{y}$	$\sigma_{y}$	$\gamma_{y}$	$\lambda_{y}$	*R*	${\hat{R}}_{\mathrm{MC}}$	Bias	RMSE
0	0.7	−0.1	0.3	0	0.5	−0.2	0.1	0.50945	0.50135	−0.00809	0.00031
0	0.7	−0.1	0.3	0.5	0.5	−0.2	0.1	0.71367	0.70749	−0.00618	0.00027
0	0.7	−0.1	0.3	0	0.7	−0.2	0.1	0.52511	0.51830	−0.00680	0.00032
0	0.7	−0.1	0.3	0.5	0.7	−0.2	0.1	0.70569	0.69953	−0.00616	0.00028
0	0.7	−0.1	0.3	0	0.5	−0.4	0.1	0.49137	0.48216	−0.00921	0.00033
0	0.7	−0.1	0.3	0.5	0.5	−0.4	0.1	0.69977	0.69122	−0.00855	0.00032
0	0.7	−0.1	0.3	0	0.7	−0.4	0.1	0.50809	0.50197	−0.00612	0.00028
0	0.7	−0.1	0.3	0.5	0.7	−0.4	0.1	0.69163	0.68582	−0.00581	0.00031
0	0.7	−0.1	0.3	0	0.5	−0.2	0.2	0.49602	0.48986	−0.00616	0.00030
0	0.7	−0.1	0.3	0.5	0.5	−0.2	0.2	0.70372	0.69768	−0.00604	0.00028
0	0.7	−0.1	0.3	0	0.7	−0.2	0.2	0.50870	0.50619	−0.00250	0.00027
0	0.7	−0.1	0.3	0.5	0.7	−0.2	0.2	0.69299	0.68945	−0.00354	0.00027
0	0.7	−0.1	0.3	0	0.5	−0.4	0.2	0.47863	0.47359	−0.00504	0.00030
0	0.7	−0.1	0.3	0.5	0.5	−0.4	0.2	0.68979	0.68290	−0.00689	0.00031
0	0.7	−0.1	0.3	0	0.7	−0.4	0.2	0.49230	0.48817	−0.00413	0.00031
0	0.7	−0.1	0.3	0.5	0.7	−0.4	0.2	0.67876	0.67517	−0.00359	0.00030

**Table 5:** Positive-shape mean, bias, and root mean squared error (RMSE) of ${\hat{R}}_{MC}$(N=1000andn=100).

$\mu_{x}$	$\sigma_{x}$	$\gamma_{x}$	$\lambda_{x}$	$\mu_{y}$	$\sigma_{y}$	$\gamma_{y}$	$\lambda_{y}$	*R*	${\hat{R}}_{\mathrm{MC}}$	Bias	RMSE
0	0.7	0.1	0.3	0	0.5	0.2	0.1	0.53183	0.52561	−0.00623	0.00031
0	0.7	0.1	0.3	0.5	0.5	0.2	0.1	0.71971	0.71151	−0.00820	0.00031
0	0.7	0.1	0.3	0	0.7	0.2	0.1	0.54076	0.53576	−0.00500	0.00031
0	0.7	0.1	0.3	0.5	0.7	0.2	0.1	0.71272	0.70567	−0.00705	0.00029
0	0.7	0.1	0.3	0	0.5	0.4	0.1	0.54736	0.54108	−0.00628	0.00031
0	0.7	0.1	0.3	0.5	0.5	0.4	0.1	0.73060	0.72414	−0.00646	0.00027
0	0.7	0.1	0.3	0	0.7	0.4	0.1	0.55478	0.54712	−0.00765	0.00030
0	0.7	0.1	0.3	0.5	0.7	0.4	0.1	0.72470	0.71812	−0.00658	0.00032
0	0.7	0.1	0.3	0	0.5	0.2	0.2	0.51767	0.51462	−0.00305	0.00029
0	0.7	0.1	0.3	0.5	0.5	0.2	0.2	0.71043	0.70328	−0.00715	0.00030
0	0.7	0.1	0.3	0	0.7	0.2	0.2	0.52393	0.52199	−0.00194	0.00032
0	0.7	0.1	0.3	0.5	0.7	0.2	0.2	0.70105	0.69579	−0.00526	0.00031
0	0.7	0.1	0.3	0	0.5	0.4	0.2	0.53280	0.52864	−0.00416	0.00031
0	0.7	0.1	0.3	0.5	0.5	0.4	0.2	0.72130	0.71402	−0.00728	0.00029
0	0.7	0.1	0.3	0	0.7	0.4	0.2	0.53775	0.53627	−0.00148	0.00028
0	0.7	0.1	0.3	0.5	0.7	0.4	0.2	0.71325	0.70860	−0.00464	0.00030

**Table 6:** Negative-shape mean, bias, and root mean squared error (RMSE) of ${\hat{R}}_{MC}$ (*N* = 10,000 and *n* = 100).

$\mu_{x}$	$\sigma_{x}$	$\gamma_{x}$	$\lambda_{x}$	$\mu_{y}$	$\sigma_{y}$	$\gamma_{y}$	$\lambda_{y}$	*R*	${\hat{R}}_{\mathrm{MC}}$	Bias	RMSE
0	0.7	−0.1	0.3	0	0.5	−0.2	0.1	0.5094	0.5021	−0.00737	0.00030
0	0.7	−0.1	0.3	0.5	0.5	−0.2	0.1	0.7137	0.7058	−0.00783	0.00030
0	0.7	−0.1	0.3	0	0.7	−0.2	0.1	0.5251	0.5198	−0.00529	0.00030
0	0.7	−0.1	0.3	0.5	0.7	−0.2	0.1	0.7057	0.6994	−0.00629	0.00030
0	0.7	−0.1	0.3	0	0.5	−0.4	0.1	0.4914	0.4841	−0.00731	0.00029
0	0.7	−0.1	0.3	0.5	0.5	−0.4	0.1	0.6998	0.6921	−0.00764	0.00029
0	0.7	−0.1	0.3	0	0.7	−0.4	0.1	0.5081	0.5022	−0.00594	0.00030
0	0.7	−0.1	0.3	0.5	0.7	−0.4	0.1	0.6916	0.6854	−0.00621	0.00029
0	0.7	−0.1	0.3	0	0.5	−0.2	0.2	0.4960	0.4908	−0.00521	0.00029
0	0.7	−0.1	0.3	0.5	0.5	−0.2	0.2	0.7037	0.6986	−0.00512	0.00028
0	0.7	−0.1	0.3	0	0.7	−0.2	0.2	0.5087	0.5059	−0.00282	0.00029
0	0.7	−0.1	0.3	0.5	0.7	−0.2	0.2	0.6930	0.6892	−0.00376	0.00028
0	0.7	−0.1	0.3	0	0.5	−0.4	0.2	0.4786	0.4728	−0.00588	0.00029
0	0.7	−0.1	0.3	0.5	0.5	−0.4	0.2	0.6898	0.6837	−0.00610	0.00029
0	0.7	−0.1	0.3	0	0.7	−0.4	0.2	0.4923	0.4885	−0.00379	0.00029
0	0.7	−0.1	0.3	0.5	0.7	−0.4	0.2	0.6788	0.6750	−0.00381	0.00028

**Table 7:** Summary statistics for the stock prices log-returns.

Data Set	Min.	1st Qu	Median	Mean	3rd Qu.	Max.	Std. dv.	Skewness	Kurtosis
BBAS3	−0.1057	−0.0097	0.0019	0.0012	0.0136	0.0736	0.0204	−0.3452	5.7413
ITUB4	−0.0492	−0.0105	0.0004	0.0006	0.0109	0.0794	0.0172	0.3809	4.4864
VALE3	−0.0689	−0.0140	0.0001	−0.0002	0.0128	0.0989	0.0231	0.4092	4.5967
VIIA3	−0.1075	−0.0344	−0.0059	−0.0030	0.0231	0.1504	0.0447	0.6144	3.6044

**Table 8:** Parameter estimates for the stock prices log-returns: BBAS4, ITUB4, VIIA3 and VALE3.

Data Set	$\hat{\lambda }$	$\hat{\mu }$	$\hat{\sigma }$	$\hat{\gamma }$
BBAS3	0.0103	−0.0063	0.0219	−0.2535
ITUB4	−0.0373	−0.0064	0.0165	−0.1545
VALE3	−0.0088	−0.0095	0.0222	−0.1631
VIIA3	−0.0058	−0.0217	0.0396	−0.1170

**Table 9:** Information criteria and Kolmogorov–Smirnov (KS) *p*-values for GEV and TGEV models.

Data Set	Distribution	AIC	BIC	EDC	KS *p*-Value
BBAS3	**TGEV**	**−1615.84**	**−1654.23**	**−1621.90**	0.0155
	GEV	−1613.83	−1642.63	−1618.38	0.0133
ITUB4	**TGEV**	**−1749.33**	**−1787.72**	**−1755.39**	0.3303
	GEV	−1747.28	−1776.07	−1751.82	0.4422
VALE3	**TGEV**	**−1556.20**	**−1594.59**	**−1562.26**	0.2388
	GEV	−1554.18	−1582.97	−1558.73	0.2313
VIIA3	**TGEV**	**−1143.34**	**−1181.73**	**−1149.40**	0.6851
	GEV	−1141.40	−1170.19	−1145.94	0.7091

**Table 10:** Stress–strength probability estimates and Bootstrap confidence interval (CI) for log-returns following TGEV distribution.

*X*	*Y*	$\hat{R}$	95% CI
VALE3	BBAS3	0.53	(0.40; 0.59)
VALE3	ITUB4	0.52	(0.40; 0.59)
VALE3	VIIA3	0.45	(0.38; 0.52)

## Data Availability

Data are available upon request.

## References

[1] Domma F., Giordano S. (2012). A stress–strength model with dependent variables to measure household financial fragility. Stat. Methods Appl..

[2] Rathie P.N., Ozelim L.d.S. (2017). Exact and approximate expressions for the reliability of stable Lévy random variables with applications to stock market modelling. J. Comput. Appl. Math..

[3] Quintino F.S., Oliveira M., Rathie P.N., Ozelim L.C.S.M., Fonseca T.A. (2024). Asset selection based on estimating stress-strength probabilities: The case of returns following three-parameter generalized extreme value distributions. AIMS Math..

[4] Kotz S., Lumelskii Y., Pensky M. (2003). The Stress-Strength Model and Its Generalizations: Theory and Applications.

[5] Bachelier L. (1900). Theorie de la Speculation, Doctor Thesis, Annales Scientifiques Ecole Normale Sperieure III-17. The Random Character of Stock Market Prices.

[6] Embrechts P., Klüppelberg C., Mikosch T. (2013). Modelling Extremal Events: For Insurance and Finance.

[7] Taleb N. (2020). Statistical Consequences of Fat Tails (Technical Incerto Collection).

[8] Jenkinson A.F. (1955). The frequency distribution of the annual maximum (or minimum) values of meteorological elements. Q. J. R. Meteorol. Soc..

[9] Cirillo P., Taleb N.N. (2016). Expected shortfall estimation for apparently infinite-mean models of operational risk. Quant. Financ..

[10] Gettinby G.D., Sinclair C.D., Power D.M., Brown R.A. (2004). An analysis of the distribution of extreme share returns in the UK from 1975 to 2000. J. Bus. Financ. Account..

[11] Goncu A., Akgul A.K., Imamoğlu O., Tiryakioğlu M., Tiryakioğlu M. (2012). An analysis of the extreme returns distribution: The case of the Istanbul Stock Exchange. Appl. Financ. Econ..

[12] Hussain S.I., Li S. (2015). Modeling the distribution of extreme returns in the chinese stock market. J. Int. Financ. Mark. Inst. Money.

[13] Nadarajah S. (2003). Reliability for extreme value distributions. Math. Comput. Model..

[14] Abbas K., Tang Y. (2014). Objective Bayesian analysis of the Frechet stress–strength model. Stat. Probab. Lett..

[15] Jia X., Nadarajah S., Guo B. (2017). Bayes estimation of *P*(*Y* < *X*) for the Weibull distribution with arbitrary parameters. Appl. Math. Model..

[16] Nojosa R., Rathie P.N. (2020). Stress–strength reliability models involving generalized gamma and Weibull distributions. Int. J. Qual. Reliab. Manag..

[17] Aryal G.R., Tsokos C.P. (2009). On the transmuted extreme value distribution with application. Nonlinear Anal. Theory Methods Appl..

[18] Nascimento F., Bourguignon M., Leão J. (2016). Extended generalized extreme value distribution with applications in environmental data. Hacet. J. Math. Stat..

[19] Otiniano C., De Paiva B., Neto D. (2019). The transmuted gev distribution: Properties and application. Commun. Stat. Appl. Methods.

[20] Rathie P.N., Ozelim L.C.d.S.M., Quintino F., Fonseca T.A.d. (2023). On the Extreme Value H-Function. Stats.

[21] Mathai A., Saxena R., Haubold H. (2009). The H-Function: Theory and Applications.

[22] Kundu D., Raqab M. (2009). Estimation of *R* = *P*(*Y* < *X*) for three-parameter Weibull distribution. Stat. Probab. Lett..

[23] R Core Team (2022). R: A Language and Environment for Statistical Computing.

